# *Rosa rugosa* Low Caloric Fiber Protein Preparations Rich in Antioxidant Flavanols and Ellagitannins

**DOI:** 10.3390/molecules28248021

**Published:** 2023-12-09

**Authors:** Joanna Milala, Monika Kosmala, Michał Sójka, Krzysztof Kołodziejczyk, Robert Klewicki, Magdalena Król, Elżbieta Karlińska, Edward Rój

**Affiliations:** 1 Institute of Food Technology and Analysis, Lodz University of Technology, B. Stefanowskiego 2/22, 90-537 Łódź, Poland; michal.sojka@p.lodz.pl (M.S.); robert.klewicki@p.lodz.pl (R.K.); krol.magdalenaa@gmail.com (M.K.); elzbieta.karlinska@p.lodz.pl (E.K.); 2 Department of Sugar Industry and Food Safety Management, Lodz University of Technology, Wólczańska 171/173, 90-530 Łódź, Poland; krzysztof.kolodziejczyk@p.lodz.pl; 3 Łukasiewicz Research Network—New Chemical Syntheses Institute, Tysiąclecia Państwa Polskiego 13a, 24-110 Puławy, Poland; edwardroj13@wp.pl

**Keywords:** rosa defatted seeds, antioxidant activity, polyphenols, ellagitannins, dietary fiber

## Abstract

Defatted seed residues after the extraction of rose oil have their potential not fully described in the existing literature. The aim of this study was to determine and characterize the components important for the human body that are found in *Rosa rugosa* defatted seeds, including dietary fibers, proteins, selected minerals, polyphenols and antioxidant activity. *Rosa rugosa* seeds defatted with CO_2_ in supercritical conditions are a rich source of dietary fibers (approx. 65%) and proteins (15%); their macronutrients include the following: Ca (175.9), Mg (83.9), K (199.2) and Na (3.5 mg/100 g). They also contain polyphenols, including flavanols (0.9%) and total ellagic acid (0.5%), and they exhibit antioxidant activity (143.8 µM TAEC/g). Tellimagrandin I and II and rugosin A were found in the extracts, and ellagitannins with a yet-indeterminate structure were also present. The seeds also contained ellagitannin derivatives—galloyl-HHDP-glucose and bis-HHDP-glucose—at the same time, and they are characterized by a low-fat content—0.4%. The energy value of defatted rose seeds is about half the energy value of popular seeds used in the food industry. The findings of the present study suggest that defatted rosehip seeds, the by-product of rosehip processing, could be an important source of bioactive components like dietary fibers, flavanols, ellagitannins and mineral compounds. Therefore, defatted rose seeds are very promising and require further research, because they can potentially be used as a natural source of chemopreventive agents.

## 1. Introduction

The rose (genus *Rosa*) includes about 120–200 species found mainly in the temperate zone of the northern hemisphere. In Europe, there are about 30 species, while 14–20 of them grow in Poland, in the wild. The probable cradle of roses is East Asia, in particular the area of China where the species varies the most. The geographical distribution of this plant varies greatly. The rose is found in natural habitats almost all over Europe, Asia Minor and North Africa. In Poland, it is often found in thickets, on the edges of forests, clearings, at the border of fields and along roads [1,2,3,4].

The wrinkled rose (*Rosa rugosa*) comes from East Asia. This species is widely cultivated, but it is also very commonly found in a feral form. The bush grows to a height of 2 m; it has thick erect stems with numerous runners. Shoots are gray, felt-hairy and densely covered with spines. The characteristic mossy pseudo-fruits of the rose are quite soft, flattened, up to 3 cm in diameter and weigh about 5 g and are orange red in color [4].

*Rosa rugosa* fruit and flakes are used in the food industry as an addition to jams, preserves, fruit purees, desserts for children and syrups. In the pharmaceutical industry, they are used for the production of vitamin preparations and herbal teas. Rosehip seeds have been evidenced to exhibit a series of biological activities such as gastrointestinal protection and diabetic, anti-aging and immunity-enhancing properties. They are the source of many ingredients, including, above all, phenolic components and mainly hydrolysable tannins [5,6,7] but also other nutrients such as fatty acids, terpenes, tocopherols, carotenoids, proteins, sugars and minerals [8,9]. Rose seeds are rich in unsaturated fatty acids (oleic acid and linoleic acid). Oils received from seeds of Rosa plants are common on the market and recommended for nutritional, cosmetic, and pharmaceutical purposes. Rosehip seeds may be offered as dietary supplements and as an additive in functional foods [10,11]. The cosmetics industry uses fruit extracts and seed oil for the production of cosmetic preparations [12], especially in preparations with anti-wrinkle properties, due to the elastase-inhibitor activity of rosehip seeds [11].

Polysaccharide-rich fractions (crude polysaccharides; CPL) from various *R. rugosa* achenes containing sugars, proteins and phenolic compounds have the ability to inhibit hyaluronidase activity. This can have an impact on the functioning of connective tissues, inflammatory processes, tumor invasion and metastasis [13].

*R. rugose* seeds can be recommended as an oil source in the skin care, cosmetic and dermatology industry thanks to their most desirable oil characteristics, in addition to being a source of the fatty acids required in terms of health [14]. In the literature, there are some data on the use of seeds after oil extraction from plants, such as camelina seeds and sophia seeds [15], grape seeds [16,17], poppy seeds [17], flaxseeds [18], blackcurrant and strawberry seeds [19], to obtain polyphenolic extracts or to fortify foods like cookies and breads. Available literature data indicate that defatted seeds of plants from the *Rosacea* family (waste after the oil extraction process) obtained from raspberries, strawberries or blackberries may be a source of many valuable nutrients and bioactive substances like dietary fibers and polyphenols [20,21,22]. These components have a beneficial pro-health effect. Polyphenols have been associated with a reduced risk of cardiovascular diseases and cancer. They have been attributed to several features, including antioxidant properties and free-radical scavenging activities [23,24]. Dietary fibers are essential for the proper functioning of the intestines, and they also lower the content of cholesterol and glucose in the blood [20,21].

Defatted seed residues after the extraction of rose oil have their potential not fully described in the existing literature. According to Moure et al. [25] and Concha et al. (2006) [26], *Rosa rubignosa* defatted residues are a source of dietary fibers, and these authors found that they can be a source of polyphenol preparations with high antioxidant activity. *Rosa rubignosa* defatted residues are a source of dietary fibers. They can be also a source of polyphenol preparations with a high antioxidant activity. So far, there are no studies on the content of bioactive ingredients such as flavanols and ellagitannins in defatted rose seeds as well as on the possibilities of the waste management of such products. That is why the aim of this study was to determine and characterize the components important for the human body, dietary fibers, proteins, selected minerals and polyphenols, in *Rosa rugosa* defatted seeds.

## 2. Results and Discussion

Defatted rose seeds were a rich source of fibers (65%) and proteins (15%); they contained moderate amounts of metabolizable carbohydrates (12%), minerals (such as ash 2.6%) and small amounts of fats (0.4%) (Table 1.). The energy value of the defatted seeds was 241 kcal/100 g on average. This value is much lower than the caloric value (energy value) of seeds used in the production of foodstuffs and/or consumed directly by humans, e.g., pumpkin seeds (446 kcal/100 g), sunflower seeds (584 kcal/100 g), sesame seeds (572 kcal/100 g) or linseeds (533 kcal/100 g) [27].

A statistical analysis showed significant differences in the composition of the tested samples obtained from different batches of the raw material. These differences may be the result of different origins of the starting material and different conditions of the de-oiling process in which individual batches of waste material were obtained. Comparing the obtained results on the composition of defatted seeds with the literature data from Kosmala et al. [20,21], it can be concluded that the dominant component, i.e., dietary fibers, is at a similar level compared to the content in defatted strawberry seeds (68.61%) and is slightly lower than that in defatted blackberry seeds (72.7%) or in defatted raspberry seeds (73.71%). Defatted rose seeds have a protein content similar to that of defatted strawberry seeds (15.62%), and it is higher than defatted blackberry seeds (10.5%) or defatted raspberry seeds (10.42%). In addition, rose seeds contain slightly more ash than defatted blackberry seeds (1.8%) or raspberries (1.81%) and, in two cases, is less than defatted strawberry seeds (3.63%).

The content of minerals showed a significant fluctuation in the samples from the individual batches. The mean content of calcium was 175.9 ± 62.1 mg/100 g, and that of magnesium was 83.9 ± 20.5 mg/100 g, that of potassium was 199.2 ± 92.2 mg/100 g and that of sodium was 3.5 ± 1.2 mg/100 g. The obtained results are consistent with the literature data. The potassium content of rose fruits, in the whole fruits and fruit parts, is very diverse. According to Kazaz at al. [28], potassium content in the damask rose was from 2243 mg/kg to 12,454 mg/kg and between 3231 mg/kg and 14,545 mg/kg in the dog rose. Demir and Özcan (2001) [29] reported a potassium level of 890.5–1023.9 mg/kg, and Ercisli [30] reported 5467 ppm and Kovacs et al. [31] 4200–11,900 ppm in various rose species. According to Kazaz at al. [28], calcium contents of the damask rose and canina rosehip fruits and fruit parts were found to be between 3885 (seeds)—11,162 (fruit flesh) mg/kg—and 3800 (seeds)—8442 mg/kg (fruit flesh). Demir and Özcan [29] reported that the Ca contents in rosehip fruits range between 133.3–146.7 ppm and Ercisli [30] reported them as 2867 ppm. The magnesium contents in the fruits and fruit parts of *Rosa damascene* and *Rosa canina* amounted to 441–2175 mg/kg, and the sodium contents ranged from 98 to 163 mg/kg [28].

Table 2 presents the antioxidant activity and content of the selected groups of polyphenols, as determined by the HPLC method, in defatted rose seeds.

The research data show that defatted rose seeds are sources of antioxidants, primarily of flavanols (801 ÷ 944 mg/100 g) and ellagic acid (326 ÷ 901 mg/100 g), bound or (in a smaller amount) free. The main representative of ellagitannins is ellagitannin calculated with a molecular weight of 1870 ((negative molecular ion peaks [M − H]^−^ at *m*/*z* 934 z = 2) (139 ÷ 291 mg/100 g)). The antioxidant capacity, measured by the DDPPH method, is 120.3–183.0 µM TAEC/g.

Particular batches of defatted seeds differed significantly in the content of ellagic acid. However, the differences in the content of flavanols were statistically insignificant. The literature data confirm the presence of the above-mentioned groups of compounds in roses. The authors of [32] state that the content of ellagitannins in *Rosa rugosa* fruits is up to 110 mg per 100 g of fresh raw material. The second dominant group of polyphenolic compounds in the obtained extracts were procyanidins. Teleszko [33] indicates that the content of procyanidin polymers in *Rosa rugosa* fruits exceeds 25,600 μg/g DM, whereas Cunja et al. [34] and Milala et al. [1] show the presence of flavonols in pseudo-rose fruits at a level of 41.2–117 mg/100 g DM, depending on the variety. Research by Koczko et al. [7] shows that Rosa species, due to their antioxidant properties, should be considered potential ingredients of functional foods The relationship (correlation) between the antioxidant activity and the content of the determined substances was examined, and it showed a significant influence of the content of the total ellagic acid and main ellagitannins on the antioxidant potential (Table 3).

A complete list of the polyphenol compounds found in defatted *Rosa rugosa* seeds is given in Table 4.

In addition to the dominant ellagitannin (negative molecular ion peaks [M − H]^−^ at *m*/*z* 934 z = 2), tellimagrandin I and II and rugosin A were also found in each of the extracts. In addition, ellagitannins with a yet-indeterminate structure were present. The extract also contained ellagitannin derivatives—galloyl-HHDP-glucose and bis-HHDP-glucose. Procyanidins were also found.

Flavonoids were present in the defatted seeds, and one of the most important glycosides was tiliroside. Free kaempferol and quercetin and glycoside derivatives of these compounds were also detected.

So far, the literature references pay little attention to *Rosa rugosa* seeds, including defatted ones. Much more attention was paid to the pseudo-fruits and flowers of plants belonging to the *Rosa* genus. Fecka [8] investigated the qualitative composition of *Rosa canina* pseudo-fruits; the presence of tellimagrandin I and II as well as monomeric and dimeric rugosines (A, B, D and E) were found. Wang et al. [39] and Olennikov et al. [40] report that among the tannins found in the *Rosa* genus, there are tellimagrandins I and II, rugosin A–E, roshenins A–E, agrimoinic acid, agrimoniin, sanguiin H-2, H-4, H-2, and H-6, casuarictin, stachuarin, lambertianin A and others. Ouerghemmi et al. [41] found that the extracts of rose leaves contain kaempferol 3-*O*-rutinoside, kaempferol 3-*O*-glucoside and keamferol 7-*O*-glucoside. The results of research conducted by the same author, Fecka [7], showed that rose seeds contain tiliroside and astragalin. As a result of research performed by Buchwald et al. [4], it was found that rosehips contain cyanidin-3-*O*-glucoside, cyanidin-3,5-diglucoside, peonidin-3-glucoside, quercetin, isoquercetin, astragalin, rutin, and also flavonoid glycosides. Mármol et al. [35] confirm that tiliroside is present in the acetone extracts of pseudo-fruits and rose seeds. Hvattumet et al. [43] confirm the presence of quercetin and its glycosides in rose fruits.

## 3. Materials and Methods

### 3.1. Material

The research material was defatted seeds of *Rosa rugosa*, obtained from Łukasiewicz-New Chemical Syntheses Institute in Puławy (Poland), after oil extraction by supercritical extraction with carbon dioxide. The supercritical fluid extraction of three batches of seeds was performed using an “ELAB” pilot plant, made at Łukasiewicz-INS, obtaining defatted seeds (SFE s. 1, SFE s. 2 and SFE s. 3). Before extraction, the seeds were grounded in a roll mill to facilitate the entrance of CO_2_ to the inner part of the seeds. Extraction was conducted under 30 MPa, at 50 °C, and the CO_2_ mass flow rate was 0.023 kg/s, and the extraction yield was 10–14%. The extraction time was 3.5 h, and the mass of the extraction material was 5 kg. The CO_2_ consumption amounted to 57.96 kg CO_2_/kg d.m. (dry mass).

### 3.2. Determination of the Nutritional Composition

Analyses of preparations: AOAC methods were used to determine the dry matter and ash, 940.26; proteins, 920.152; crude fats, 930.09; and total dietary fiber (TDF), 985.29 [45]. The metabolized carbohydrate content (MC) and the energy value (EV) were calculated using the formulas below [46]:

“MC” = “100%−”(P + F + A + DF + W)

“EV” = “MC”⋅4 + P⋅4 + F⋅9 + DF⋅2

where P = protein content [%]; F = fat content [%]; A = ash content [%]; DF = dietary fiber content [%]; W = water content [%].

### 3.3. Mineral Content

The contents of elements were determined as previously described by Sójka et al. [47]. The samples were calcined in a furnace at 550 °C for 16 h. The ash was then solubilized in 1 mol/L HNO_3_, and minerals were analyzed by the atomic absorption spectroscopy (AAS) method (SOLAAR 969 AAS spectrometer, Unicam, Ilminster, UK).

### 3.4. HPLC-PDA Measurement of Polyphenols

Polyphenol (free ellagic acid and main ellagitannin) content: The extraction of polyphenols was performed with the following solution: acetone:water:formic acid (70:29.9:0.1 *v*/*v*/*v*). A total of 500 mg of the material was mixed with 4 mL of a solvent and was sonified for 15 min. Next, the mixture was centrifuged at 14,000× *g*, and the solution was decanted. This procedure was repeated twice with 3 mL of solvents. Extracts were transferred to a 10 mL measuring flask and were filled up with solvents.

Total ellagic acid: A total of 100 mg of the material, 0.5 mL of 70% glycerol and 75 µL of concentrated TFA were poured into a 2 mL flask, then mixed and hydrolyzed for 18 h at 95 ± 1 °C. Then, the sample was quantitatively transferred into a 5 mL measuring flask and filled up with methanol [48].

The polyphenols and total ellagic acid were analyzed in the HPLC Smartline (Knauer, Berlin, Germany) chromatograph with a PDA detector. A Phenomenex Gemini 5u C18 110a (250 × 4.60 mm; 5 μm, Phenomenex, Torrance, CA, USA) column was kept at 35 °C, and the flow rate was 1.25 mL/min at an injection volume of 20 μL. Phase A was 0.05% (*v*/*v*) phosphoric acid in water and phase B was 83/20/17 (*v*/*v*/*v*) acetonitrile/methanol/water. The gradient was as follows: 0–5 min, 4% of phase B; 5–12.5 min, 4–15% of phase B; 12.5–42.5 min, 15–40% of phase B; 42.5–51.8 min, 40–50% of phase B; 51.8–53.4 min, 50–55% of phase B; and 53.4–55 min, 4% of phase B. Detection conditions were as follows: 360 nm for ellagic acid and 250 nm for ellagitannins. Ellagic acid and ellagitannin agrimoniin were used as standards. The method parameters are described in [44].

### 3.5. HPLC-FD Measurement of Flavanols

Proanthocyanidin degradation in an acidic environment with an overdose of phloroglucinol were performed according to Sójka et al. [48]: a total of 20 mg of the sample was mixed with 800 μL of a methanol solution containing phloroglucinol (75 g/L) and ascorbic acid (15 g/L). Phloroglucinolysis was started by adding 400 μL of 0.2 mol/L of hydrochloric acid in methanol and lasted for 30 min at 50 °C, and then the reaction was stopped by adding 600 μL of a 40 mmol/L sodium acetate solution and by being placed in an ice bath. The samples were centrifuged for 5 min at 12,000× *g* and were diluted with a 40 mmol/L sodium acetate solution. A chromatograph with a fluorescence detector (FD) RF-10AXL (Shimadzu, Tokyo, Japan) equipped with a Phenomenex Gemini 5u C18 110a (250 × 4.60 mm; 5 μm) column was used. The gradient was 0–10 min, 4−7% phase B; 10–27 min, 7–30% phase B; 27–29 min, 30–70% phase B; 29–34 min, 70% phase B; 34–35 min, 70–4% phase B; and 35–40 min, 4% phase B. Phase A consisted of a 2.5% water solution (*v*/*v*) of acetic acid, and phase B consisted of 80% (*v*/*v*) acetonitrile in water. The flow rate was 1 mL/min, and the temperature was 25 °C. The identification was conducted using the following standards: (−)-epicatechin, (+)-catechin, (−)-epicatechin-phloroglucinol and (+)-catechin-phloroglucinol. Quantitative analyses of the released flavonols, namely, (+)-catechin and (−)-epicatechin and phloroglucinol adducts, were conducted on the basis of chromatograms recorded with the FD set at a 278 nm excitation wavelength and a 360 nm emission wavelength. The method parameters are described in [49].

### 3.6. Qualitative Measurement of Selected Polyphenols by UHPLC-DAD-MS

Ultra-High-Performance Liquid Chromatography and Electrospray Ionization Mass Spectrometry (UHPLC-ESI-MS): A Dionex UltiMate 3000 UHPLC apparatus (Thermo Fisher Scientific, Waltham, MA, USA) coupled with a Thermo Scientific Q Exactive quadrupole ion trap mass spectrometer was used. A Phenomenex Luna 5 μm C18 column (250 × 4.6 mm) was used. Phase A was formic acid in water (1/99 *v*/*v*), and phase B was acetonitrile\methanol\water (63/20/17 *v*/*v*). The gradient was 0–6 min, 5% of phase B; 6–36 min, 5–28% of phase B; 36–48 min, 28–73% of phase B; 48–54 min, 73% of phase B; 54–60 min, 73–5% of phase B; and 60–70 min, 5% of phase B. The DAD detector recorded spectra simultaneously in the range of 200–600 nm, and the mass spectrometer recorded spectra in negative mode (H-ESI source). The source parameters were set as follows: vaporizer temperature, 500 °C; ion spray voltage, 4 kV; and capillary temperature, 400 °C; the sheath gas and auxiliary gas flow rates were 75 and 20 units, respectively. In full MS/dd-MS2 scanning mode, the mass range was set at *m*/*z* 200–2000 and the collision energy at 20 eV. Data were collected using the Xcalibur software 3.063 (Thermo Fisher Scientific, Waltham, MA, USA). The results of the polyphenol identification are given in Table 4.

### 3.7. Antioxidant Activity

Antioxidant activity was measured by the DPPH method and calculated as Trolox equivalent (TEAC) [50].

### 3.8. Statistics

All analyses were performed in duplicates. The test results were subjected to a statistical analysis using the one-way analysis of variance. For the obtained results, Duncan’s post-hoc test was performed at a significance level of α ≤ 0.05. The calculations were performed in the Statistica 10 software (Stat Soft, Tulsa, USA).

## 4. Conclusions

Rose seeds defatted with CO_2_ in supercritical conditions are a rich source of dietary fibers (approx. 65%) and proteins (15%). At the same time, they are characterized by a low-fat content—0.4%. Moreover, the seeds are a source of macronutrients, including Ca (175.9), Mg (83.9 mg/100 g), K (199.2 mg/100 g) and Na (3.5 mg/100 g). Defatted in supercritical conditions, rose seeds show antioxidant activity (143.8 µM TAEC/g) and contain polyphenols, including flavanols (0.9%) and total ellagic acid (0.5%). Tellimagrandin I and II, rugosin A and sanguiin H-6 with a gallic group were found in the extracts, and ellagitannins with a yet-indeterminate structure were present. The extract also contained ellagitannin derivatives—galloyl-HHDP-glucose and bis-HHDP-glucose. The energy value of defatted rose seeds is about half the energy value of popular seeds used in the food industry. The findings of the present study suggest that defatted rosehip seeds, the by-product of rosehip processing, could be an important source of bioactive components as dietary fibers, flavanols, ellagitannins and mineral compounds. Therefore, defatted rose seeds are very promising and require further research, because they can potentially be used as a natural source of chemopreventive agents.

There is a need for further research to determine the biological effect of defatted rose seeds as well as the possibility of obtaining valuable polyphenol extracts.

## Figures and Tables

**Table 1:** Chemical composition of seeds defatted with CO2 under supercritical conditions.

Defatted Seeds	SFE s.1	SFE s.2	SFE s.3	Mean
Dry matter	95.6 ± 0.0 b	95.6 ± 0.1 b	93.3 ± 0.1 a	94.8 ± 1.3
Ash %	2.4 ± 0.1 b	3.5 ± 0.1 c	1.9 ± 0.0 a	2.6 ± 0.6
Ca mg/100 g	152.0 ± 5.7 b	246.4 ± 4.8 c	129.3.0 ± 4.6 a	175.9 ±62.1
Mg mg/100 g	69.6 ± 2.6 a	107.4 ± 1.7 b	74.8 ± 0.9 a	83.9 ± 20.5
K mg/100 g	125.2 ±10.8 a	302.5 ± 7.1 c	170.0 ± 0.8 b	199.2 ± 92.2
Na mg/100 g	3.0 ± 0.4 a	4.9 ± 0.2 b	2.6 ± 0.1 a	3.5 ± 1.2
Protein %	15.1 ± 0.3 a	15.0 ± 0.4 a	14.1 ± 0.1 b	14.7 ± 0.5
Dietary Fibers %	66.1 ± 0.3 b	61.2 ± 0.4 a	67.4 ± 0.1 c	64.9 ± 2.7
Metabolized carbohydrates, %	11.3 ± 0.2 b	15.6 ± 0.2 c	9.7 ± 0.1 a	12.2 ± 2.5
Fat %	0.6 ± 0.1 b	0.3 ± 0.1 a	0.1 ± 0.0 a	0.4 ± 0.2
Energy value, kcal/100 g	243.5 ± 0.2 b	247.8 ± 0.6 c	231.1 ± 0.4 a	240.8 ± 7.1

The same letters in the line are not statistically significant differences with *p* < 0.05.

**Table 2:** The antioxidant activity and content of selected groups of polyphenols in defatted rose seeds.

Polyphenols	SFE s.1	SFE s.2	SFE s.3	Mean
Main ellagitannin mg/100 g	146.5 ± 7.3 a	270.5 ± 11.3 b	138.5 ± 0.7 a	185.2 ± 74.0
Free ellagic acid mg/100 g	12.1 ± 1.7 a	33.1 ± 0.0 b	11.7 ± 0.4 a	19.0 ± 12.2
Total ellagic acid mg/100 g	452.9 ± 20.9 b	900.7 ± 59.6 c	325.7 ± 22.9 a	559.8 ± 302.0
Flavanols mg/100 g	801.2 ± 12.4 a	943.8 ± 83.4 a	851.9 ± 12.2 a	865.6 ± 72.3
Antioxidant activity DPPH µM TAEC/g	128.0 ± 6.3 a	183.0 ± 10 b	120.3 ± 2.0 a	143.8 ± 34.2

The same letters in the line are not statistically significant differences with *p* < 0.05.

**Table 3:** The relationship (correlation) between the antioxidant activity and the content of the determined substances.

Variable	The Spearman Rank-Order Correlation Coefficient Significant Differences *p* < 0.0500			
	Total Ellagic Acid	Free Ellagic Acid	Main Ellagitanin	Flavanols
Antioxidant activity DPPH	0.885714	0.771429	0.942857	0.371429

**Table 4:** Qualitative composition of polyphenolic compounds in defatted *Rosa rugosa* seeds’ identification [7,35,36,37,38,39,40,41,42,43,44].

	RT [min]	MS Data [*m*/*z*]	MS/MS Data	MW [g/mol]	Compound
**1**	16.5	[783.07]^−^	783, 451, 301	784	Bis-HHDP-glucose
		[633.07]^−^	633, 301	634	HHDP-galloyl-glucose
**2**	18.2	[783.07]^−^	783, 451, 301	784	Bis-HHDP-glucose
		[633.07]^−^	633, 301	634	HHDP-galloyl-glucose
**3**	20.7	[577.14]^−^	425, 408, 407, 289, 137, 125	578	Procyanidin dimer
		[785.09]^−^	483, 419, 313, 301	786	di-*O*-galloyl-HHDP-glucose Tellimagrandin
**4**	21.65	[865.2]^−^	695, 577, 407, 287, 125	866	Procyanidin trimer
**5**	22.6	[289.07]^−^	245, 241, 125	290	Catechin
**6**	24.5	[1153.26]^−^	983, 865, 695, 577, 449, 287, 125	1154	Procyanidin tetramer
**7**	26.1	[785.09]^−^	633, 483, 419, 301	786	di-*O*-galloyl-HHDP-glucose Tellimagrandin I
**9**	28.56	[635.09]^−^	465, 313, 295, 223, 211, 193, 169, 125	636	Tri-*O*-galloyl-glucoside
**10**	29.1	[1153.26]^−^	983, 865, 695, 577, 449, 287, 125	1154	Procyanidin tetramer
**11**	29.4	[933.07]^−^	631, 451, 301	934	Ellagitannin
		[865.2]^−^	695, 577, 543, 451, 425, 407, 287,125	866	Procyanidin trimer
**12**	30.55	[935.08]^−^	898, 765, 633, 463, 301	936	Galloyl bis HHDP- glucose
**13**	32.8	[1018.0]^2−^	1691, 1567, 1265, 1209, 1059, 935, 897, 783, 633, 301	2038	Ellagitannin
**14**	33.8	[937.1]^−^	785, 767, 741, 635, 465, 419, 301	938	Tri-galloyl-HHDP-glucose Tellimagrandin II
**15**	34.01	[1105]^−^	1061, 937, 917, 909, 891, 805, 785, 767, 749, 633, 615, 597, 465, 425, 301, 275, 169, 125	1106	Rugosin A
**16**	35.39	[787.1]^−^ [935]^2−^	635, 617, 483, 465, 447, 313, 295 1569, 1085, 1059, 935, 917, 787, 767, 633, 451, 301	788 1872	Tetragalloyl glucose Ellagitannin
**17**	36.12	[934]^2−^	1567, 1265, 1085, 935, 897, 783, 633, 301	1870	Ellagitannin
**18**	37.08	[615.1]^−^	463, 301	616	Quercetin-galloylhexoside
		[433.04]^−^	301	434	Ellagic acid pentoside
**19**	38.02	[1085.08]^−^	783, 633, 613, 451, 301	1086	Galloyl-castalagin
**20**	38.4	[301.0]^−^		302	Ellagic acid
**21**	39.02	[463.09]^−^	301	464	Quercetin hexoside
**22**	39.73	[939.11]^−^	769, 617, 599, 465, 447, 431, 169	940	Pentagalloylglucose
**23**	39.99	[477.1]^−^	477, 313, 265, 169	478	not identified
**24**	40.04	[1085.08]^−^	783, 633, 613, 451, 301	1086	Galloyl-castalagin
**25**	41.53	[433.08]^−^	433, 301, 300	434	Ellagic acid pentoside
**26**	42.45	[447.09]^−^	301	448	Quercetin 3-rhamnoside
**27**	46.13	[593.13]^−^	463, 285	594	Tiliroside *
**28**	46.3	[301.04]^−^		302	Quercetin
**29**	48.6	[285.04]^−^		286	Kaempferol

* kaempferol-3-*O*-β-d-(6″-*E*-*p*-coumaroyl)-glucopyranoside.

## Data Availability

The data presented in this study are available on request from the corresponding author. The data are not publicly available due to due to the volume of the partial data.
